# Investigating diagnostic potential of long non-coding RNAs in head and neck squamous cell carcinoma using TCGA database and clinical specimens

**DOI:** 10.1038/s41598-024-57987-y

**Published:** 2024-03-29

**Authors:** Ting Lan, Yuxiang Yan, Dali Zheng, Lincan Ding

**Affiliations:** 1 Fujian Key Laboratory of Oral Diseases, Fujian Biological Materials Engineering and Technology Center of Stomatology, School and Hospital of Stomatology, Fujian Medical University, 88 Jiao Tong Road, Fuzhou, 350004 Fujian China; 2 Department of Preventive Dentistry, School and Hospital of Stomatology, Fujian Medical University, 246 Yang Qiao Middle Road, Fuzhou, 350000 Fujian China

**Keywords:** LncRNA, TCGA, HNSCC, Biomarker, Cancer genomics, Head and neck cancer, Tumour biomarkers

## Abstract

Head and neck squamous cell carcinoma (HNSCC) is a prevalent and prognostically challenging cancer worldwide. The role of long non-coding RNAs (lncRNAs) in cancer regulation is progressively being understood. This study aims to identify lncRNAs with diagnostic potential as biomarkers for HNSCC. Statistical analysis was performed on expression data from the Cancer Genome Atlas (TCGA) database to identify potential lncRNAs associated with HNSCC. Four selected lncRNAs were validated using real-time quantitative reverse transcription polymerase chain reaction and correlated with clinical factors. Functional roles were further investigated. A total of 488 differentially expressed lncRNAs were identified in TCGA-HNSC. After rigorous evaluation based on p-values, survival analysis, and ROC analysis, 24 lncRNAs were prioritized for additional investigation. LINC00460, LINC00941, CTC-241F20.4, and RP11-357H14.17 were established as candidate diagnostic biomarkers. These lncRNAs exhibited elevated expression in HNSCC tissues and were associated with poor prognosis. Combining them showed high diagnostic accuracy. Notably, LINC00460 and CTC-241F20.4 demonstrated a significant elevation in the advanced stages of HNSCC. We constructed an lncRNA-mRNA regulatory network, and the array of significant regulatory pathways identified included focal adhesion, regulation of epithelial cell migration, and others. Additionally, these lncRNAs were found to influence immune responses by modulating immune cell infiltration in the HNSCC microenvironment. Our research indicates that LINC00460, LINC00941, RP11-357H14.17, and CTC-241F20.4 may have diagnostic and prognostic importance in HNSCC. Furthermore, we have gained insights into their potential functional roles, particularly about immune responses and interactions in the microenvironment.

## Introduction

HNSCC, also known as head and neck squamous cell carcinoma, ranks as the sixth most prevalent cancer globally, causing approximately 930,000 new instances and 460,000 fatalities in 2020^1^. The prognosis for HNSCC still heavily relies on histopathological diagnosis and tumor stage. The slight increase in survival rates witnessed in the last thirty years, going from 55 to 66%, is primarily credited to the rise of HPV-related HNSCC, which carries a more favorable outlook, rather than advancements in multimodality therapy^2^. Nevertheless, due to the diversity of HNSCC, individuals with comparable medical characteristics might experience varying treatment results^3^. Predicting the outcome of HNSCC remains challenging, and molecular testing has yet to influence the selection of HNSCC treatment^4^. Hence, it is essential to identify diagnostic and prognostic indicators to guide treating patients with HNSCC.

A long non-coding RNA (lncRNA) refers to an RNA molecule consisting of over 200 nucleotides, which does not contain genetic information for protein synthesis but instead performs regulatory functions in gene expression across epigenetic, transcriptional, and post-transcriptional stages^5^. The dysregulated manifestation of lncRNAs has been linked to the onset and advancement of different types of tumors, such as HNSCC, emphasizing their crucial function in cancer biology^6–9^. The potential of lncRNA signatures obtained from normal and cancer tissues as a novel category of biomarkers has been demonstrated. However, the roles of only a few dysregulated lncRNAs in HNSCC have been thoroughly understood. Among the lncRNAs examined in HNSCC^10^, some of them include HOTAIR, HOTTIP, UCA1, LET, MEG3, MALAT1, H19, and NAG7. For example, the increase in MALAT1 expression has been demonstrated to control the epithelial-mesenchymal transition (EMT) process and enhance the invasion and migration of tumor cells by regulating N-cadherin, Vimentin, and E-cadherin^11^. LncRNAs, originating from 17,948 loci, have been identified in recent human genome annotation (GRch38, GENCODE Release 37; accessed on 15 February 2021), with a total of 48,741 transcripts. Nevertheless, the precise function of numerous lncRNAs in HNSCC is still not well comprehended and necessitates additional exploration^12,13^.

This study utilized data analysis from The Cancer Genome Atlas (TCGA) to identify possible lncRNA molecules in HNSCC patients. Afterward, we confirmed these results by performing real-time qRT-PCR on paired HNSCC and neighboring healthy tissues. Our analysis identified LINC00460, LINC00941, CTC-241F20.4, and RP11-357H14.17 as potential oncogenic genes in this study.

## Materials and methods

### Data collection and processing from the TCGA database

We acquired transcriptome sequencing data from the TCGA-HNSC project. This data consists of HTSeq-FPKM values for gene expression levels, and HTSeq counts for differentially expressed genes (DEGs), as well as the corresponding clinical data. Utilizing the R package TCGAbiolinks facilitated the accomplishment of this task^14^. The dataset comprised 502 HNSCC samples and 44 para-tumor tissues. We collected clinical data, including tumor grade, TNM stage, follow-up duration, HPV status, and survival outcome.

Our attention was directed towards 14,855 long non-coding genes from the downloaded data, encompassing diverse biotypes such as “3prime overlapping lncRNA", "antisense", "macro lncRNA", "lincRNA", "noncoding", "processed transcript", "sense intronic", and "sense overlapping". The HTSeq-FPKM data underwent a log2 transformation after adding 0.01 to facilitate comparison. Subsequently, low-expressed lncRNAs, defined as those with fewer than one count per sample, were filtered out for further analyses.

### Detection of LncRNAs with altered expression

For the differential expression analysis using DESeq2, a log2-fold change cutoff of |3| and a p-value cutoff of 0.05 were both utilized. Volcano plots and heat maps were used to visualize the expression patterns of the differentially expressed lncRNAs.

### Survival outcome analysis

Patients with HNSCC were categorized into low and high-expression groups based on the median expression level of each lncRNA. Statistically significant p-values less than 0.05 were considered using the R package survival to perform univariate Cox analysis. In order to further evaluate the impact of candidate lncRNAs and the riskscore on predicting the prognosis of patients with HNSCC, we conducted a Kaplan–Meier (KM) survival analysis to compare the overall survival rates among various groups of patients. The analysis was conducted utilizing the R packages survival and survminer, which assisted in identifying the most suitable threshold. We conducted a multivariate Cox analysis utilizing the R package survival to assess further the risk score model based on the candidate lncRNAs.

### Receiver operating characteristic (ROC) analysis

The accuracy of diagnostic and prognostic indicators was assessed by generating ROC curves with the R package pROC. We evaluated the discrimination and diagnostic accuracy of lncRNAs by constructing ROC curves and determining the area under the receiver operating characteristic curve (AUC). During this examination, individuals without the disease were regarded as controls, whereas patients with HNSCC were classified as positive data sets. LncRNAs with an AUC value exceeding 0.8 were chosen as potential candidate genes. Furthermore, the model's efficiency was assessed using a time-based ROC curve.

### Clinical patient samples

Between September 2019 and November 2020, the Department of Oral and Maxillofacial Surgery at the First Hospital of Fujian Medical University collected 42 head and neck squamous carcinoma samples, along with corresponding healthy tissues. Normal tissues that matched were defined as those located at least 5 cm away from the unaffected edge of the tumor. This study received approval from the biomedical research ethics committee of the stomatological hospital affiliated with Fujian Medical University (approval number: 2021-FMUSS-034), which included relevant details of the experimental protocol. Surgically obtained samples were ground and stored at − 80 °C before being used for RNA and DNA extraction. All methods were carried out according to relevant guidelines and regulations.

### Extraction of RNA and performing real-time quantitative reverse transcription polymerase chain reaction (qRT-PCR)

The Trizol RNA extraction method (Trizol, Vedbaek, Denmark) was used to extract RNA from the clinically acquired tissue samples first crushed in liquid nitrogen. The Prime Script kit (Takara Bio, China) was utilized for reverse transcription. Subsequently, SYBR Green (Takara Bio, China) and the primers mentioned in Table 1 were utilized for conducting real-time qRT-PCR. LncRNA expression levels were standardized using RNA18s as an internal reference. The relative quantification of lncRNAs was determined using the comparative 2^−ΔΔCT^ approach.

**Table 1:** The sequences of primers used are the following:

Gene symbol	Gene id	Primer sequences L	Primer sequences R
LINC00941	ENSG00000235884.3	TTCTTGGACTTCTCAGCCTCCA	TCTGGTAGTTGGACGGTTGCTT
RP5-884M6.1	ENSG00000228742.8	AGCAATTCATCAGAGGCCAGGA	GCTGTTTGTGGAGGTGTGGATG
LINC00460	ENSG00000233532.4	GGATGAGAACGAAGGTTACGAC	ACATAAATCGGGGTGACTTCAG
RP11-357H14.17	ENSG00000272763.1	AAACTCTCCCCAACTTCACAAA	GAGACCTACTGAGCCCTCTTCA
RP11-397A16.1	ENSG00000267284.1	TACCTCAGATGGAAATGCAGAA	GTGGGTGTTAAAGTCTCAATGG
RP5-1011O1.2	ENSG00000232498.1	TCCAGTGGCTCATCGTTACCTG	GACAACGAGGACTTGGAGGGAA
RP11-445F12.1	ENSG00000277268.1	CTCCAGCGGTGCGAAGATGATA	GATGAGTGGGGAAACGGCATTG
RP11-221N13.3	ENSG00000256268.1	TGTGCATGGAGACTAGAAATGG	TGCCTTGTGGAATGAGATAAGA
RP11-25I15.3	ENSG00000257114.2	CTTCCCCTCTCTGGGACTTAAT	TTCTATGTGGTGTCTCCCTGTG
ANO1-AS2	ENSG00000254417.1	GTGAGGCTGAAGTGTCTCCCTT	CTCAAGTCACCTTCAGCCCCTC
CTD-2377D24.6	ENSG00000244649.3	TGCAGCACATTTAATTGGAAGT	GCCTGATTTGAAGTCTTCTGCT
AC073130.1	ENSG00000237870.5	GAGGATCTGACATGAAAAGTGAC	TTTAGTACTCCCTTGGCAACCT
HOXB-AS4	ENSG00000242207.1	CTCTCCTGCCCTGACTTTGCA	TCCAGCAAGTACCCGGCAATAA
CASC9	ENSG00000249395.2	AATCAGCGAGACTCCGTGGG	TTCTTGCCAGGTGTTGTTCTGC
RP11-54H7.4	ENSG00000275216.1	CAGCCCCAAGTCATTTTCTAAC	GAGCCATCTTCTCTGCTCCTTA
CTC-480C2.1	ENSG00000250874.1	GGCAAAAGTAGCATCAAAAAGC	TAGCCCCTTCAACAAAAACACT
CTC-241F20.4	ENSG00000268186.1	GCGTGTCCGTATAGAAGACCA	CTGCATTCTCCCACAGTAGAAA
RP11-493L12.3	ENSG00000257925.1	ACATAAGTCAGCTCCAGATCGAC	TCCGTGGACATGCACAGGATA
LINC01468	ENSG00000231131.5	CTGAAGACGGAGCCCTGAGAC	GGTCACAGCATCCCAGAAGG
RP11-215P8.4	ENSG00000250564.1	TGGCTGAAAATGGAGATGAAGAG	GGCCTTTCAATTTTCAAAGCAGT
LINC01179	ENSG00000249500.1	CTGGTCTCAAACTCCTGACCTC	TGTGTGCCCTCCATGAATATAA
LINC00973	ENSG00000240476.1	TCTGTGGGAGAAGTAGGTGGTT	CCAGGGAAAGAGATAACAATGC
LINC00925	ENSG00000255571.5	CCTGAAATTGTCCTGTGAAGTG	AGGCTACTTGTTCTCCCTTCCT

### DNA extraction and HPV DNA detection

Genomic DNA extraction was performed using a DNA isolation kit (TIANGEN, Cat#DP304-02) per the product manual. The quality of the isolated DNA was assessed using beta- (β-) globin PCR with suitable primers, and all samples tested positive for β-globin. Subtype-specific primers TS16-A/TS16-B and TS18-A/TS18-B were used to detect HPV subtypes 16 and 18 (Table 2), as outlined in the study^15,16^. Melting curves allowing the semiquantitative assessment and the differentiation between strong positive (+ +), positive( +), or negative (−) viral load were obtained at 30 and 35 cycles^17^.

**Table 2:** The sequences of primers used for the β-globin gene, HPV genotype 16 and 18.

Target gene	Primer name	Sequence of primer
β-globin	PCO3	ACACAACTGTGTTCACTAGC
β-globin	PCO4	CAACTTCATCCACGTTCACC
HPV 16	TS16-A	GGTCGGTGGACCGGTCGATG
HPV 16	TS16-B	GCAATGTAGGTGTATCTCCA
HPV 18	TS18-A	CCTTGGACGTAAATTTTTGG
HPV 18	TS18-B	CACGCACACGCTTGGCAGGT

### LncRNA–mRNA co-expression analysis and functional enrichment analysis

LncRNA–mRNA co-expression analysis was performed to evaluate the potential roles of lncRNAs. The Spearman correlation coefficients between the lncRNAs and genes were calculated. Co-expressed genes of the lncRNAs were identified with correlation coefficients higher than 0.4 or lower than -0.4 and p-value < 0.05. Kyoto Encyclopedia of Genes and Genomes (KEGG) Pathway and gene ontology (GO) function enrichment analyses in three functional ontologies—namely, biological process (BP), cellular component (CC), and molecular function (MF)—were performed with the co-expressed genes using the R package clusterProfiler.

### Examining the association between candidate LncRNAs and levels of 28 immune cell types in tumor infiltration

Each sample's tumor-infiltrating levels of various immune cell types were computed using the R package GSVA and the single-sample gene set enrichment analysis (ssGSEA) technique. The analysis relied on a panel of immune genes that defines 28 different types of immune cells^18^. The determination of relative levels of immune cell tumor infiltration was achieved by quantifying the expression profiles of genes related to the immune system in each tumor sample.

### Statistical analysis

Statistical analyses were conducted using various software packages, including IBM SPSS Statistics 26 for Windows (URL: https://www.ibm.com/analytics/spss-statistics-software), GraphPad Prism version 9 (URL: https://www.graphpad.com/scientific-software/prism/), and the R statistical package version 4.0.2 (URL: http://www.R-project.org) as mentioned earlier. A p-value below 0.05 was deemed to have statistical significance.

### Ethics approval

All patients were informed of the purpose of specimen collection according to the guidelines of the biomedical research ethics committee of the stomatological hospital affiliated with Fujian Medical University (approval number: 2021-FMUSS-034).

### Consent to participate

Informed consent was obtained from all individual participants included in the study.

## Results

### Identification of LncRNAs with altered expression in HNSCC patients using the TCGA database

**Figure 1:**
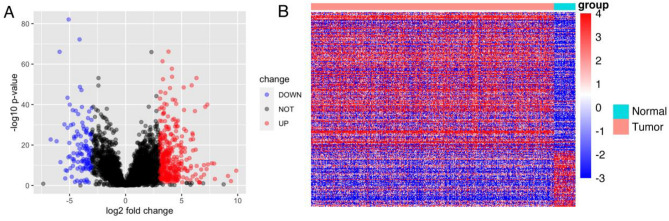
A comparison of TCGA-HNSC patients' LncRNA expression. (**A**) The volcano plots exhibited the contrasting expression of lncRNAs in HNSC tissues compared to normal tissues. LncRNAs were categorized into various color groups depending on their absolute average log2 fold change and p-value. Red indicated genes that are upregulated, while blue indicates genes that are downregulated. (**B**) LncRNA expression patterns were shown in a heat map.

The TCGA database contained 502 HNSCC samples and 44 samples of normal tissue. The detailed clinicopathological data and general information of the individuals from the TCGA database were listed in Table 3. Among these samples, there were 14,855 lncRNAs for each. Following excluding genes exhibiting low expression levels, 7915 lncRNAs were employed for subsequent analysis. We conducted differential analysis on HNSCC lncRNAs using the R package DESeq2, identifying 368 lncRNAs that were upregulated and 120 lncRNAs that were down-regulated. This analysis was based on the criteria of a p-value < 0.05 and an absolute log2 fold change > 3. The findings were depicted in a volcano plot (Fig. 1A). Figure 1B displayed the expression patterns of these lncRNAs in HNSCC tissues and normal tissues, indicating their potential contribution to the development of HNSCC.

**Table 3:** The detailed clinicopathological data and general information of the individuals from the TCGA database (middle) and Fjmu cohort (right).

Characteristic	TCGA	Fjmu
Gender		
Male	368	14
Female	133	28
Age		
50 or less	88	11
51–64	237	23
65 or more	175	8
Pathologic T stage		
T1	45	1
T2	133	10
T3	96	8
T4	171	21
Pathologic M stage		
M0	187	38
Mx	61	/
Pathologic N stage		
N0	172	24
N1	65	5
N2	164	12
N3	7	
Pathologic stage		
I	25	1
II	71	5
III	78	8
IV	259	26
HPV status (P16 testing)		
Negative	72	Not available
Positive	30	Not available
HPV status (ISH testing)		
Negative	64	Not available
Positive	19	Not available
HPV16		
Negative	Not available	17
Positive/strong positive	Not available	25
HPV18		
Negative	Not available	15
Positive/strong positive	Not available	27

### Evaluating the predictive significance of differentially expressed LncRNAs in HNSCC patients using the TCGA database

Univariate Cox proportional hazard regression analysis was performed on TCGA-HNSC patients to assess the prognostic significance of the identified lncRNAs. The individual assessment was conducted for each lncRNA with differential expression, and patients were categorized into two groups according to the expression levels of each lncRNA, either above or below the median. The analysis revealed a significant correlation between patient survival and 69 differentially expressed lncRNAs. The list of these lncRNAs can be found in supplementary Excel 1.

### The ROC curve for discriminating expression LncRNAs

The 69 lncRNAs mentioned earlier underwent ROC analysis to determine their differential expression. Supplementary Excel 2 contained a list of the AUC values for each lncRNA. Of all these lncRNAs, LINC00941 displayed the ROC curve's highest AUC value of 0.928. After applying a threshold of > 0.8 for the AUC, a total of 24 genes were chosen for further examination. These genes included LINC00941, RP5-884M6.1, LINC00460, RP11-357H14.17, RP11-397A16.1, RP5-1011O1.2, RP11-445F12.1, RP11-221N13.3, RP11-25I15.3, ANO1-AS2, CTD-2377D24.6, AC073130.1, HOXB-AS4, CASC9, RP11-54H7.4, CTC-480C2.1, CTC-241F20.4, RP11-493L12.3, LINC01468, RP11-215P8.4, LINC01179, AC009262.2, LINC00973, and LINC00925.

### Confirmation of differential expression LncRNAs in patients with HNSCC

**Figure 2:**
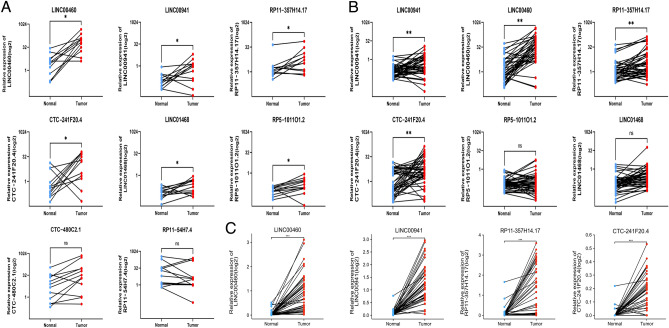
The levels of expression of the potential LncRNAs in clinical tissues of HNSCC. (**A**) In the preliminary validation, we detected eight lncRNAs in most of the 12 paired validation samples. LINC00460, LINC00941, RP11-357H14.17, CTC-241F20.4, LINC01468, and RP5-1011O1.2 displayed notable variations in expression (p < 0.05), whereas CTC-480C2.1 and RP11-54H7.4 exhibited no statistically significant differences in expression (p > 0.05). (**B**) The number of collected clinical samples was raised to 42 pairs, and we monitored the expression patterns of LINC00941, LINC00460, RP11-357H14.17, CTC-241F20.4, RP5-1011O1.2, and LINC01468. Out of these, LINC00460, LINC00941, RP11-357H14.17, and CTC-241F20.4 displayed notable variations in expression (p < 0.05), whereas RP5-1011O1.2 and LINC01468 exhibited no statistically significant differences (p > 0.05). (**C**) In the TCGA-HNSC database, LINC00460, LINC00941, RP11-357H14.17, and CTC-241F20.4 exhibited notable variations in expression levels (p < 0.05).

The 24 chosen lncRNAs underwent an initial validation using 12 sets of HNSCC tissues and their corresponding paracancerous tissues obtained from the clinic. The levels of expression of these lncRNAs were detected using real-time qRT-PCR. Among the 24 lncRNA candidates, eight were detectable in the majority of tissue samples and were utilized for statistical analysis (Fig. 2A). Out of these, six lncRNAs (LINC00941, LINC00460, RP11-357H14.17, CTC-241F20.4, RP5-1011O1.2, and LINC01468) showed notable variations in expression (p < 0.05). In contrast, the expression levels of RP11-54H7.4 and CTC-480C2.1 did not demonstrate statistical significance (p > 0.05). The expression trends of these six lncRNAs were further validated in an expanded sample size of 42 pairs of clinical samples (Fig. 2B). The clinical characteristics and general information of these 42 samples, called the Fjmu cohort, were listed in Table 3. The findings from the bioinformatic analysis were confirmed as the levels of LINC00941, LINC00460, RP11-357H14.17, and CTC-241F20 expression were markedly elevated in HNSCC tissues according to the results in TCGA database (Fig. 2C). This implied that these four lncRNAs with differential expression might contribute to the development of HNSCC and could be utilized as diagnostic indicators. The chromosomal location, strand location, transcriptional start position, transcriptional end position, and transcript size of each lncRNA were provided in Table 4.

**Table 4:** LncRNAs along with their gene symbol, chromosomal location (chrom), strand location (strand), the transcriptional start position (txStart), transcriptional end position (txEnd), and transcript size.

GeneSymbol	Chrom	Strand	txStard	txEnd	Transcript size (bp)
LINC00460	13q33.2	+	106,374,477	106,377,791	1522
LINC00941	12p11.21	+	30,795,681	30,800,008	632
CTC-241F20.4	19q13.33	−	48,262,900	48,271,283	437
RP11-357H14.17	17q21.32	−	48,635,923	48,647,023	1270

### Value of LINC00460, LINC00941, RP11-357H14.17, and CTC-241F20.4 as a joint diagnostic index

**Figure 3:**
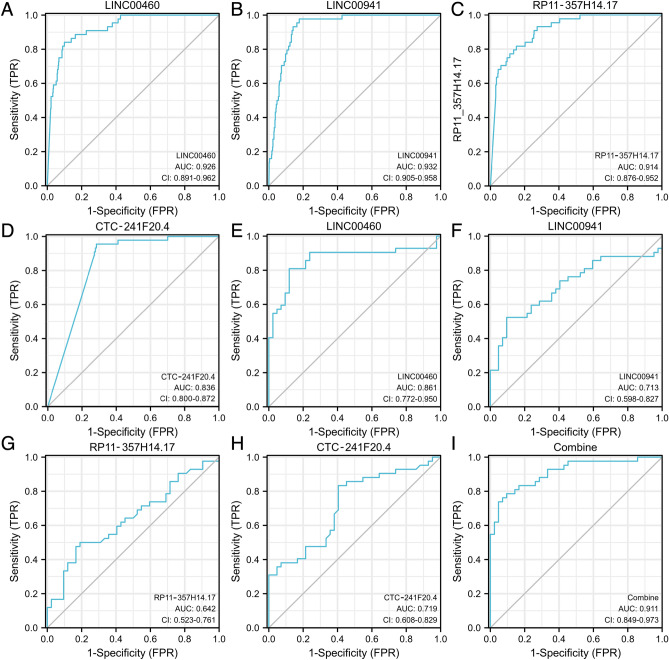
Classification accuracy of LINC00460, LINC00941, RP11-357H14.17, and CTC-241F20.4 in different datasets. (**A**–**D**) The TCGA dataset showed the classification accuracy of LINC00460, LINC00941, RP11-357H14.17, and CTC-241F20.4. (**E**–**H**) Classification accuracy of LINC00460, LINC00941, RP11-357H14.17, and CTC-241F20.4 in 42 paired clinical HNSCC tissues. (**I**) Classification accuracy of the combined use of LINC00941, LINC00460, RP11-357H14.17, and CTC-241F20.4 in 42 paired clinical HNSCC tissues.

ROC analysis was conducted on the TCGA database to evaluate the diagnostic significance of the four lncRNAs (LINC00460, LINC00941, RP11-357H14.17, and CTC-241F20.4). ROC curves were generated for each lncRNA individually (Fig. 3A–D), and the corresponding AUC values were computed. When used individually, the AUC values for LINC00460, LINC00941, RP11-357H14.17, and CTC-241F20.4 were 0.861, 0.713, 0.642, and 0.719, respectively, in clinic tissues (Fig. 3E–H). Sensitivities and specificities were also calculated for each lncRNA. In addition, the four lncRNAs were combined to generate a multifactorial diagnostic ROC curve (Fig. 3I). The combined diagnostic index had an AUC value of 0.911, demonstrating a sensitivity of 72.6% and a specificity of 92.9%. The collective diagnostic capacity of the four lncRNAs was determined to surpass that of individual lncRNA indicators, indicating their potential as efficient diagnostic biomarkers for HNSCC.

### Investigating the relationship between LINC00460, LINC00941, RP11-357H14.17, CTC-241F20.4, and the tumor stages and TMN stages in TCGA-HNSC

**Figure 4:**
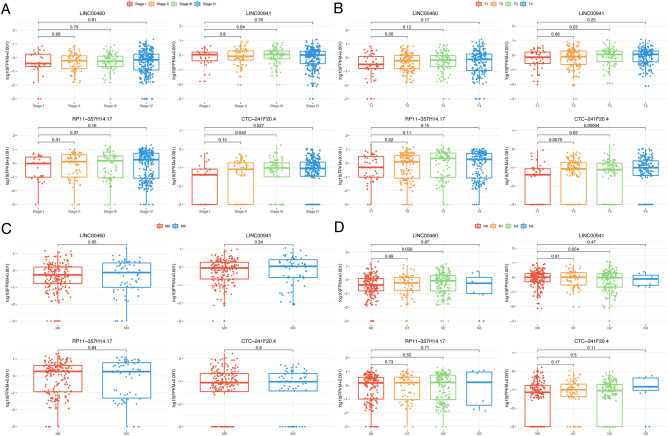
The relationship between the levels of expression of four LncRNAs and the clinical characteristics in TCGA-HNSC. The Wilcoxon test was used to evaluate the correlation between the expression levels of the four lncRNAs and various clinic features in TCGA-HNSC, including (**A**) tumor stage (stage I, stage II, stage III, and stage IV), (**B**) T stage includes (T1, T2, T3, and T4), (**C**) stage M (M0 and MX), and (**D**) stage (N0, N1, N2, and N3) of N. Stage I, T1, M0, and N0 were designated as the control group for comparison.

We utilized the Wilcox test method to examine the correlation between the expression levels of LINC00460, LINC00941, RP11-357H14.17, CTC-241F20.4, and the advancement of HNSCC in the TCGA database. Clinic features were depicted in Fig. 4, showing the variations in expression levels of these four lncRNAs. An increase in expression levels was noticed in both the tumor stage and T stage, while no correlation was found between the M stage and N stage. Compared to stage I as the control group, CTC-241F20.4 demonstrated a noteworthy increase in stage III (p = 0.043) and stage IV (p = 0.027). CTC-241F20.4 exhibited significant upregulation in T2 (p = 0.0079), T3 (p = 0.03), and T4 (p = 0.00064) when compared to T1, which served as the control group. Furthermore, in the comparison of various N stages with N0 serving as the reference group, LINC00460 exhibited noteworthy upregulation in N2 (p = 0.039). The findings from the Wilcox examination and the illustration in Fig. 4 suggested that in HNSCC tissues, particularly in later stages, these four lncRNAs, notably LINC00460 and CTC-241F20.4, tended towards elevated expression levels. This implied their possible functional involvement in the advancement of HNSC.

### Studying the correlation between LINC00460, LINC00941, CTC-241F20.4, RP11-357H14.17, and the survival outcome in TCGA-HNSC

**Figure 5:**
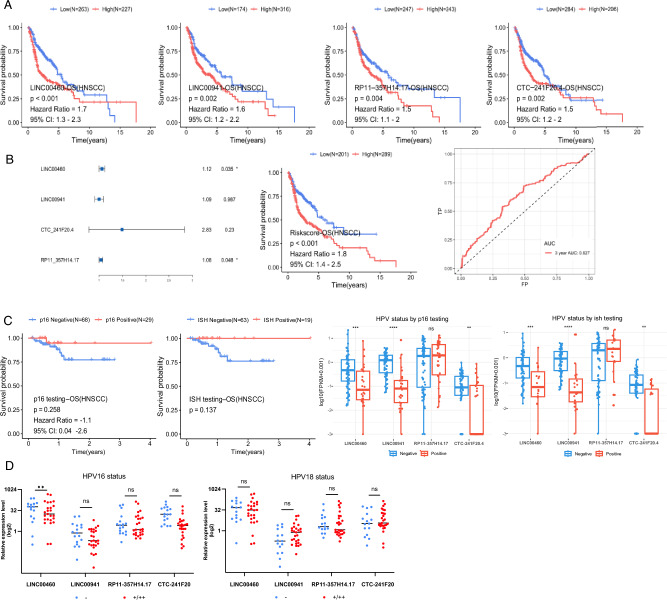
The TCGA-HNSC database contained valuable prognostic information on LINC00460, LINC00941, CTC-241F20.4, and RP11-357H14.17. (**A**) Individually, the prognostic Kaplan–Meier curves of LINC00460, LINC00941, CTC-241F20.4, and RP11-357H14.17 were analyzed. (**B**) The riskscore calculated by a multivariate Cox proportional hazards regression analysis of LINC00460, LINC00941, CTC-241F20.4, and RP11-357H14.17 can be visualized through the forest plot, Kaplan–Meier curves, and AUC (area under the curve) curves. (**C**) The prognostic significance of HPV status, determined by p16 and ISH testing, was evaluated using Kaplan–Meier survival curves. Furthermore, the expression levels of specific lncRNAs were analyzed across various HPV status groups. (**D**) Correlation between HPV status and four lncRNA expression levels in Fjmu clinical tissues.

In order to further examine the clinical importance and potential predictive ability of the four lncRNAs, we performed a Kaplan–Meier survival analysis on the TCGA-HNSC dataset using the R package survminer, explicitly employing the minimum p-value approach for grouping (Fig. 5A). The findings indicated that elevated levels of the four lncRNAs were linked to unfavorable survival results (p < 0.01). The risk ratios (RRs) for LINC00460, LINC00941, CTC-241F20.4, and RP11-357H14.17 were 1.7 (1.3–2.3, 95% confidence interval), 1.54 (1.2–2.2, 95% confidence interval), 1.5 (1.2–2.2, 95% confidence interval), and 1.5 (1.1–2.22, 95% confidence interval), respectively.

Additionally, a multivariate analysis using Cox proportional hazards regression was conducted to assess the potential correlation between the four lncRNAs combination and survival. For LINC00460, LINC00941, CTC-241F20.4, and RP11-357H14.17, the forest map revealed noteworthy p-values of p = 0.005 through the likelihood ratio test, p = 0.0009 through the Wald test, and p = 0.0009 through the log-rank statistical test. Significant expression levels of LINC00460 (p = 0.035) and RP11-357H14.17 (p = 0.048) in the model signature were associated with survival outcomes. The model signature's risk score was determined by multiplying 0.05676675 with LINC00460, subtracting 0.0007523397 multiplied by LINC00941, adding 0.3957223 multiplied by CTC-241F20.4, and adding 0.03740536 multiplied by RP11-357H14.17.Significantly different survival rates were observed due to the risk score, with the high-risk group being linked to unfavorable survival outcomes. Figure 5B demonstrated that the predictive accuracy of the 3-year AUC was moderately satisfactory in the time-dependent ROC analysis, reaching 0.627.

### Investigating the relationship between LINC00460, LINC00941, CTC-241F20.4, RP11-357H14.17, and HPV infection status

Several studies suggest that HPV infection may impact the prognosis of HNSCC patients. Analysis of TCGA data on HNSCC cases found that patients who tested negative for HPV using p16 or ISH methods had lower survival rates, consistent with previous research^19,20^. Investigation into the association between four lncRNAs and HPV testing indicated elevated expression levels of LINC00460, LINC00941, and CTC-241F20.4 in HPV-negative patients with a poorer prognosis (Fig. 5C). The tumor samples from the Fjmu cohort underwent HPV genotyping, with specific primers for HPV16 and HPV18 utilized (Table 3 and Supplementary Excel 3). Among the 42 samples, 25 tested positive for HPV16 (with 3 showing stronger positivity) and 17 tested negative. Likewise, 27 samples tested positive for HPV18 (with 3 showing stronger positivity), and 15 samples tested negative. In addition, the analysis of the relationship between the expression levels of four lncRNAs and HPV status suggested that these four lncRNAs exhibited a trend of higher expression levels in HPV-negative tissues, especially in patients with LINC00460 subtype negative HPV16, where the expression levels are higher with statistical significance (p < 0.01, Fig. 5D).

### Integrative analysis of lncRNA and mRNA expression profiles and functional enrichment analysis

**Figure 6:**
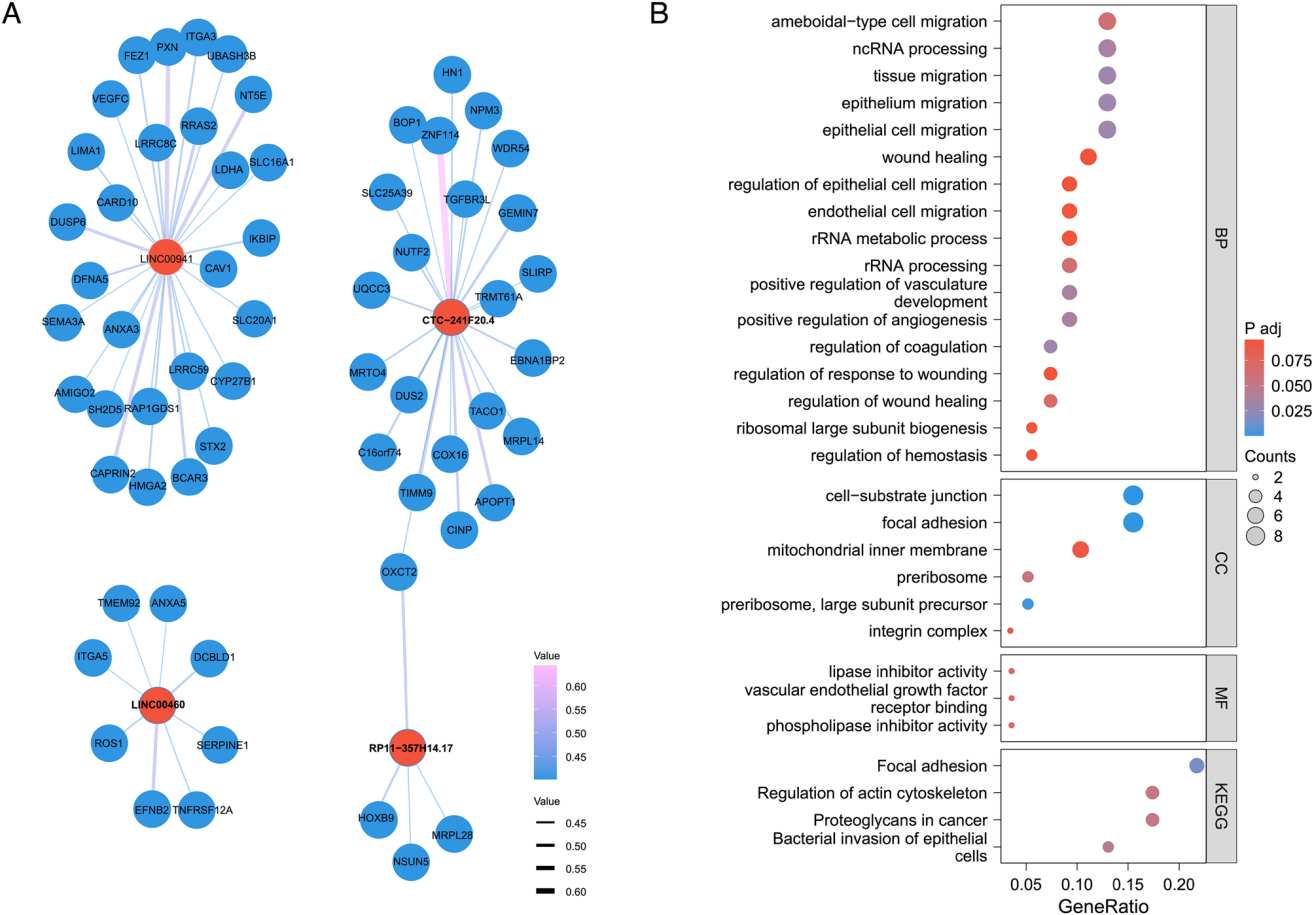
Prediction of the 4 LncRNA function. (**A**) A total of 63 mRNAs co-expressed with LINC00460, LINC00941, CTC-241F20.4, and RP11-357H14.17 were selected based on Spearman correlation coefficients from the expression profiles in the TCGA-HNSC dataset. (**B**) GO and KEGG pathway enrichment analyses were performed for these 63 co-expressed mRNAs to determine their potential functions.

We constructed an lncRNA-mRNA regulatory network to explore the regulatory mechanisms through an integrative analysis of expression profiles within the TCGA-HNSC dataset. We identified co-expressed genes for the lncRNAs with correlation coefficients higher than 0.4 or lower than -0.4, accompanied by a p-value of less than 0.05. This analysis yielded a list of 63 co-expressed mRNAs, detailed in supplementary material four and illustrated in Fig. 6A.

We conducted GO and KEGG enrichment analyses in pursuit of a comprehensive understanding of the 4 lncRNA functional pathway. Our findings indicated that 37 pathways were significantly enriched, as detailed in supplementary material 5. We showcased a total of 30 enriched pathways, inclusive of all findings from MF, CC, and KEGG categories, as well as the principal 17 BP results, depicted in Fig. 6B. The array of significant regulatory pathways identified included focal adhesion, bacterial invasion of epithelial cells, proteoglycans in cancer, regulation of the actin cytoskeleton, wound healing, and regulation of epithelial cell migration, among others.

### The TCGA-HNSC database showed a correlation between LINC00460, LINC00941, CTC-241F20.4, RP11-357H14.17, and levels of tumor infiltration

**Figure 7:**
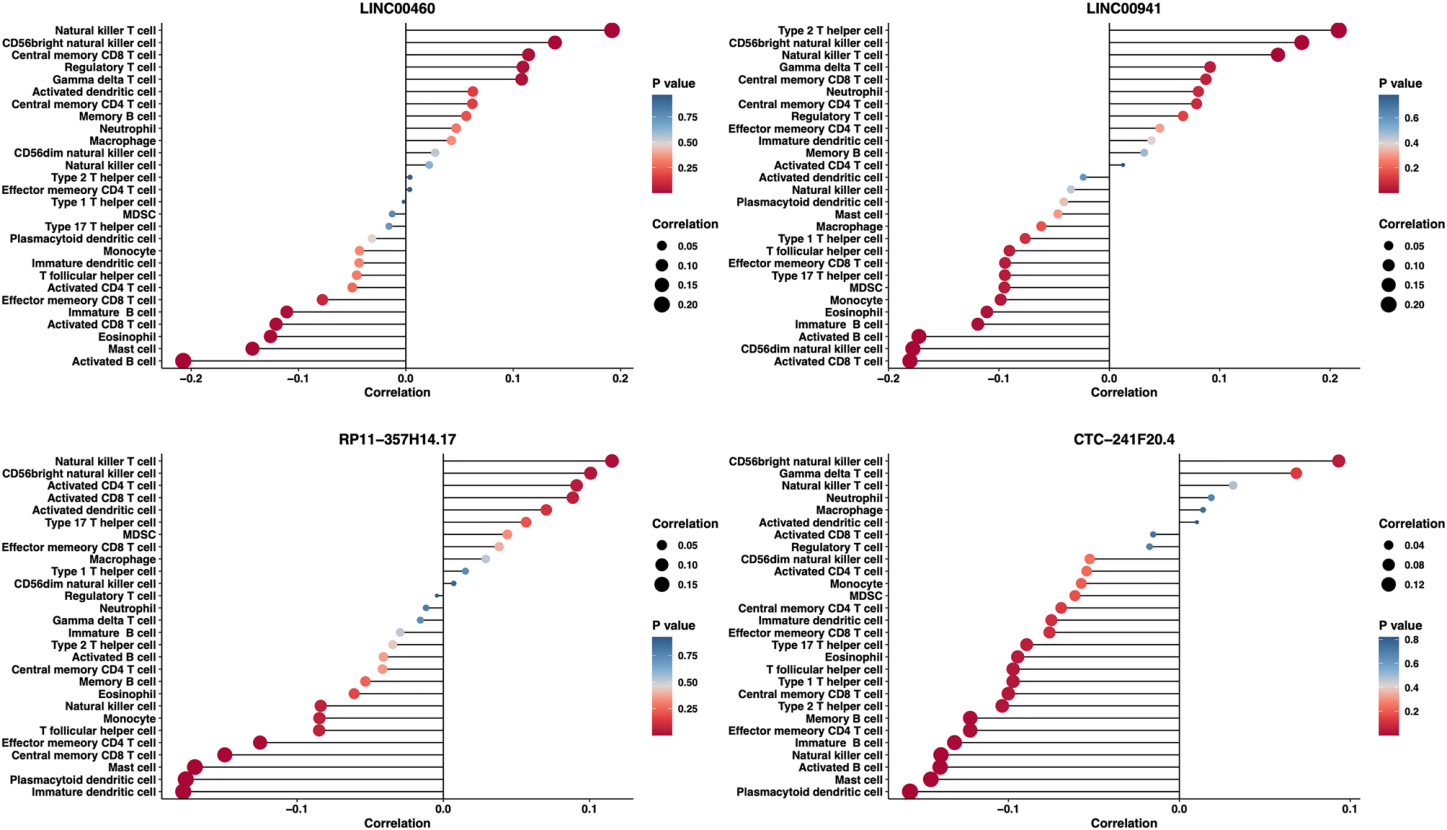
The correlation between the four LncRNAs and infiltration of the immune system. The lollipop chart demonstrated the connection between the expression levels of LINC00460, LINC00941, CTC-241F20.4, and RP11-357H14.17 and the infiltration of 28 subsets of immune cells. The Spearman R value indicates the level of correlation between lncRNAs and immune cell infiltration, represented by the size of the dots.

In order to examine the influence of the four lncRNAs on the tumor microenvironment of HNSCC, their connection with the quantities of various immune cell categories was assessed through ssGSEA, and the Spearman correlation coefficients were computed. The lollipop plot (Fig. 7) demonstrated that the levels of LINC00460, LINC00941, CTC-241F20.4, and RP11-357H14.17 were positively associated with CD56 bright natural killer cells (R = 0.1389, R = 0.1743, R = 0.1007, R = 0.0933; p < 0.05). Supplementary Excel 6 provided a list of the connections between LINC00460, LINC00941, CTC-241F20.4, RP11-357H14.17, and the 28 diverse types of immune cells, indicating that these four lncRNAs impact the infiltration of immunocytes into the microenvironment of HNSCC.

## Discussion

A thorough analysis of selective constraints reveals that approximately 87% of the transcribed regions in the human genome produce non-functional RNAs, often called 'junk' RNAs. It is worth mentioning that these transcripts are not simply discarded materials but play a crucial role in developing lncRNAs^21^. LncRNAs play a role in controlling gene expression through interactions with DNA, RNA, and proteins across various stages. Modulating chromatin structure and function impacts RNA splicing, stability, and translation^22^. Given the multifaceted nature of gene regulation, it is not surprising that dysregulated lncRNAs have been linked to cancer. The discovery suggests that lncRNAs have great potential as valuable indicators and targets for treating different forms of cancer^23^. Nevertheless, our knowledge regarding lncRNAs specifically associated with HNSCC is still restricted^10^.

For our current investigation, we examined the TCGA dataset to discover lncRNAs expressed differently in HNSCC. A comprehensive screening process involving p-value ranking, survival analysis, and ROC screening allowed us to detect 488 lncRNAs in HNSCC. Afterward, we confirmed the expression levels for 24 chosen lncRNAs in clinical tissues. In this study, we identified the expression levels of 8 lncRNAs in 12 initial pairs of HNSCC tissues. These lncRNAs include LINC00941, LINC00460, RP11-357H14.17, CTC-241F20.4, RP5-1011O1.2, LINC01468, RP11-54H7.4, and CTC-480C2.1. However, most of the remaining lncRNAs were undetectable in the corresponding paracancerous tissues and a subset of HNSCC tissues. Despite the upregulation of these candidate lncRNAs in a few HNSCC tissues, statistical analysis was not feasible due to the limited sample size. Following validation using 42 pairs of clinical tissues, we identified four candidate lncRNA markers (LINC00460, LINC00941, CTC-241F20.4, and RP11-357H14.17) that displayed significant diagnostic value for HNSCC patients.

Afterward, we generated ROC curves for these four potential lncRNAs utilizing the TCGA-HNSC database. All four curves demonstrated AUC values exceeding 0.8, indicating their discriminatory solid power. Moreover, the diagnostic potential of the four candidate lncRNAs was evaluated by conducting ROC analysis on 42 pairs of HNSCC clinical tissues. The AUC values for each lncRNA were as follows: 0.861 (95% CI 0.772–0.950; p < 0.0001) for LINC00460, 0.713 (95% CI 0.598–0.827; p = 0.0008) for LINC00941, 0.642 (95% CI 0.523–0.761; p = 0.025) for CTC-241F20.4, and 0.719 (95% CI 0.608–0.829; p = 0.0006) for RP11-357H14.17. These lncRNAs exhibited sensitivities ranging from 47.6% to 83.3% and specificities ranging from 59.5 to 90.5%. Since no single lncRNA or miRNA has demonstrated significant diagnostic value on its own, combining multiple biomarkers proves to be more efficacious^24,25^. Consequently, we generated a multifactorial combined diagnostic ROC curve for the four lncRNAs (Fig. 3I). This resulted in an AUC value of 0.911 (95% CI 0.849–0.973; p < 0.0001), demonstrating a sensitivity of 72.6% and specificity of 92.9%. The combined diagnostic ability of these four lncRNAs proved to be superior and could serve as potentially effective diagnostic biomarkers for HNSCC.

Realizing the significant diagnostic capability of these four potential lncRNAs, we analyzed their associations with clinical features and levels of tumor infiltration in the TCGA-HNSC database. In HNSCC, the levels of all four lncRNAs were discovered to be increased. During our analysis, we examined the associations between LINC00460, LINC00941, RP11-357H14.17, CTC-241F20.4, clinical features, and tumor infiltration levels in the TCGA-HNSC dataset. In HNSCC tissues, we noticed increased expression of these four lncRNAs, which directly correlated with the stage and T stage. In advanced stages, LINC00460 and CTC-241F20.4 showed increased expression, indicating their potential functional significance in HNSCC, as shown in Fig. 4. We have constructed an lncRNA-mRNA co-expression network and performed functional enrichment analysis of 63 co-expression genes closely correlated with lncRNA expression. Our findings, which suggested a potential regulatory network involving lncRNAs and a series of significant pathways such as focal adhesion and regulation of epithelial cell migration, hint at a potential link with cellular migration and adhesion. Furthermore, we discovered that these four lncRNAs impacted immune responses by affecting the infiltration of immune cells in the microenvironment of HNSCC (Fig. 7).

Earlier studies on HNSCC have documented the capacity of specific lncRNAs to identify the condition. For example, the excessive expression of MALAT1 has been linked to the occurrence and unfavorable prognosis of HNSCC^11,26,27^. UCA1 regulates cell proliferation, and its overexpression has been linked to lymph node involvement^28,29^. This study aims to identify novel biomarkers with potential for future research, specifically focusing on lncRNAs. We have chosen a broad array of lncRNA candidates, as opposed to a limited selection, and have corroborated our findings through validation in independent cohorts. As a result, we have identified LINC00460, LINC00941, RP11-357H14.17, and CTC-241F20.4 as potential biomarkers for the diagnosis and prognosis of HNSCC. Previous studies in HNSCC have shown that LINC00460 enhances EMT, proliferation, and differentiation by aiding the entry of PRDX1 into the nucleus^30^. The overexpression of LINC00941 has been observed in tissues and cells of oral squamous cell carcinoma, contributing to the progression of diffuse-type gastric cancer by stimulating cell proliferation, migration, invasion, and metastasis through the induction of EMT and activation of the Wnt/β-catenin pathway^31^. The gene RP11-357H14.17 plays a role in the formation of new blood vessels, development, and specialization of cells in endometrial cancer, and it is strongly linked to unfavorable prognosis for patients^32^.

The TCGA-HNSC database provided extensive clinical and lncRNA sequencing data resources, so we utilized them to overcome sample size limitations. To summarize, our investigation indicates that these four lncRNAs have the potential to be utilized as diagnostic and prognostic indicators in HNSCC. Additionally, their collective assessment using clinical tissues could aid in diagnosis. Nevertheless, utilizing novel lncRNA biomarkers will encounter obstacles and possible concerns. The statistical significance may be affected due to the restricted quantity of clinical samples and incomplete clinical survival information in this study. The expression levels of these four lncRNAs showed a significant correlation with adverse clinical features such as HPV status and immune infiltration. Furthermore, lncRNA-mRNA co-expression analysis indicated their association with multiple critical cellular functional pathways. However, it is imperative to conduct functional investigations in cell lines or panels in order to gain a deeper understanding of the involvement of these potential lncRNAs in the advancement of HNSCC and their possible prognostic importance for patients.

## Conclusions

Throughout this investigation, we extensively examined the TCGA database and clinical samples of HNSCC. Through various analyses, including survival analysis, gene expression level analysis, and ROC analysis, we identified four candidate lncRNA biomarkers: LINC00460, LINC00941, CTC-241F20.4, and RP11-357H14.17. The lncRNAs showed increased expression in HNSCC tissues and were linked to unfavorable prognosis and high accuracy in classification, especially when considering their collective diagnostic capability. Significantly, LINC00460 and CTC-241F20.4 exhibited elevated levels of expression in the later stages and HPV-negative statuses within the TCGA-HNSC dataset. Furthermore, these lncRNAs have been found to influence the regulation of cellular migration and adhesion, as well as the entry of immune cells into the microenvironment of head and neck squamous cell carcinoma.

## Supplementary Information

Supplementary Information 1.

Supplementary Information 2.

Supplementary Information 3.

Supplementary Information 4.

Supplementary Information 5.

Supplementary Information 6.

Supplementary Information 7.

## Data Availability

All datasets used in the study came from the TCGA-HNSC project, which is accessible to the public (https://portal.gdc.cancer.gov/projects/TCGA-HNSC). Please contact the corresponding author to access the data.
