# Supplementary material for: Investigating diagnostic potential of long non-coding RNAs in head and neck squamous cell carcinoma using TCGA database and clinical specimens
Source: Sci Rep. 2024 Mar 29;14:7500. doi: 10.1038/s41598-024-57987-y (PMC10980800; doi:10.1038/s41598-024-57987-y)
Supplement: Supplementary file 7 — Supplementary Information 7. [file 41598_2024_57987_MOESM7_ESM.docx]

**Supplementary Materials**

The supplementary materials provided alongside the study contain additional information, organized in Excel spreadsheets for comprehensive analysis and review. These materials include survival analysis results and AUC results of 69 differentially expressed lncRNAs, HPV16 and HPV18 Genotyping results in the Fjmu Cohort, co-expressed mRNA correlation analysis results, gene enrichment outcomes, and the association between LINC00460, LINC00941, CTC-241F20.4, and RP11-357H14.17 and tumor-infiltrating levels in the TCGA-HNSC database.
